# Intermediate-temperature electrolysis of energy grass *Miscanthus sinensis* for sustainable hydrogen production

**DOI:** 10.1038/s41598-018-34544-y

**Published:** 2018-11-01

**Authors:** Masaya Ito, Tetsuya Hori, Shinya Teranishi, Masahiro Nagao, Takashi Hibino

**Affiliations:** 1 Graduate School of Environmental Studies, Nagoya University, Nagoya, 464-8601 Japan; 2 SOKEN Inc., Nisshin, Aichi 470-0111 Japan

## Abstract

Biohydrogen produced from the electrolysis of biomass is promising because the onset voltages are less than 1.0 V and comparable to those of water and alcohol-water electrolysis. The present study focuses on *Miscanthus sinensis* as a model grass because of its abundance and ease of cultivation in Japan. The electrochemical performance and hydrogen formation properties of electrolysis cells using grass as a biohydrogen source were evaluated at intermediate temperature to achieve electrolysis. The components, such as holocellulose, cellulose, lignin, and extractives, were separated from *Miscanthus sinensis* to understand the reactions of *Miscanthus sinensis* in the electrolysis cell. The relatively high resistivity and low current-voltage performance of an electrolysis cell using lignin were responsible for degradation of the electrolysis properties compared to those with pure cellulose or holocellulose as biohydrogen resources. Biohydrogen was formed according to Faraday’s law and evolved continuously at 0.1 A cm^−2^ for 3,000 seconds.

## Introduction

The uncertain availability of crude oil has been causing not only an increase in oil costs but also uncertainty of future global energy supplies. Newly developed and/or unharnessed energy sources are thus required to realize less dependence on oil and to facilitate a transition from reliance on conventional oil sources. Among the alternatives, biomass is a potential energy source because it is commonly available, inexpensive, and a sustainable and renewable resource. Wood is placed at the top of the biomass list; however, the fundamental uses such as construction wood, firewood, and pulp should be considered and balanced to avoid excessive concentration on fuel use. Considering the 50 to 1,500 EJ yr^−1^ of biomass potential through the world in 2050^1^, 200 to 500 EJ yr^−1^ of that was estimated to be a sustainable biomass source if all agricultural lands were changed to biomass production^2,3^. Nevertheless, most biofuels are derived from crops or oilseeds, which results in competition between food and fuel uses^4^. The ongoing destruction of rainforests also highlight the limits of wood utilization. Researchers have turned their attention to lignocellulosic biomass such as perennial grasses and waste woods for biofuel production^5–8^. These represent non-edible whole-plant biomass. For example, gathered and pelletized biomass can be burned directly as a fuel for thermal plants or as a heat source^9^. Biomass can also be gasified, liquefied, and pyrolyzed to syngas and bio-oil for distributed use as household and industrial fuels. Many grasses are non-edible, so that food security risks such as “food versus fuel” can be avoided as long as the plant cultivation system does not affect the surrounding ecosystems. Grasses as a lignocellulosic material thus have potential as a next generation bioenergy resource; however, biofuel production techniques such as biochemical, thermochemical, and chemical conversion are limited by lignin, which inhibits these reactions due to the rigid cross-linking in the support tissues^10^. Biochemical conversion is the most common method for the generation of biofuels such as bio-hydrogen, ethanol, and methane. Anaerobic fermentation, thermal gasification, and pyrolysis have been studied for biohydrogen production; however, these techniques require improvements in terms of the hydrogen production rate, energy consumption, and cost including pretreatment of the biomass materials^11^. Gasification using supercritical water has also been proposed to realize higher hydrogen concentrations^12^. Microbial electrolysis has also been reported, where electrochemical reaction promoted by microbial action can produce hydrogen through the electrolysis of exudates at lower cell voltages than that of water electrolysis^13^. The electrochemical characteristics of cellulose electrolysis have also been reported using cellulose as a model biomass^14^, newspaper^15^, woody sawdust^14^, and waste biomass^16^ at around 150 °C. Cellulose in acidic solvent was supplied to the electrolysis cell and electrolyzed at a relatively higher temperature than room temperature, which is common for water electrolysis, and the onset voltage for electrolysis started from ca. 0.3 V. This result offers the possibility of direct electrolysis of lignocellulose from low cell voltages, which would contribute to a reduction of power consumption. If unused and waste plant biomass including stems, husks, and leaves of crops are applicable to electrolysis, then the obtained biohydrogen could become an alternative to hydrogen produced from the electrolysis of water. In this report, *Miscanthus sinensis* (*M. sinensis*) was considered as a model grass biomass because of its abundance and ease of cultivation in Japan. *M. sinensis* can be found in a wide area between Okinawa, a southern island and Hokkaido, the northern island of Japan^17^. This is the first report on the electrochemical performance of electrolysis cells using grasses as a hydrogen source. To reveal the characteristics of *M. sinensis* electrolysis, holocellulose, cellulose, lignin, and extractives derived from *M. sinensis* were applied as biohydrogen sources and the electrolysis performance is discussed in detail. If sufficient *I-V* performance and theoretical hydrogen production could be achieved, then this would ensure the position of unused grasses as a biomass material and as an alternative to other biofuel resources.

## Results

### *Miscanthus sinensis* as a hydrogen source

**Figure 1:**
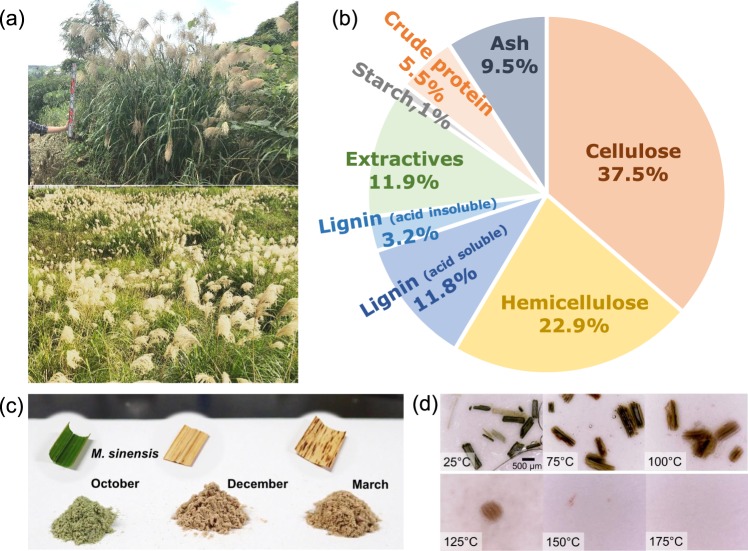
*M. sinensis* as a biomass material. (**a**) Appearance of *M. sinensis* (upper) and clustered *M. sinensis* (lower). Components of (**b**) *M. sinensis* and (**c**) milled samples collected in October, December, 2017, and March, 2018. (**d**) Photographs of the morphology change during heat treatment in phosphoric acid.

*M. sinensis* is a very common plant found from the south to the north of Japan^18,19^. Figure 1(a) shows *M. sinensis* growing wild on a vacant lot and along the roadside. The plant height is dependent on the season, latitude, and age; however, it sometimes reaches over 2 meters, especially in temperate regions such as Okinawa, where the mean annual air temperature is 23.1 °C. The components of *M. sinensis* were analyzed and are shown in Fig. 1(b). The percentage of each component is shown on a dry basis. Holocellulose (cellulose and hemicellulose) occupies 58%, which is comparable to other lignocellulosic biomass materials such as switchgrass (60–70%), wheat straw (58–69%), and rice straw (52.2–60.6%)^20^. Weeds generally consist of 50–70% holocellulose, which indicates weeds including *M. sinensis* have potential to be used as cellulosic biomass materials. Klason lignin and acid-soluble lignin, which have relatively low conversion to heat or hydrogen than cellulose, account for 15% of *M. sinensis*^21^. Ash, which occupies 9%, mainly consists of low-crystallinity silica. Except for ash, the components of *M. sinensis* have hydrogen in their chemical composition and thus potential to produce hydrogen through electrolysis. As typical examples of grasses other than *M. sinensis*, *Pueraria lobata* (*P. lobata*) and *Solidago altissima* (*S. altissima*), which represent climbing plants and perennial plants, respectively, were examined to compare the difference between the species (Fig. S1). Figure 1(c) shows the appearance of milled *M. sinensis* and those powders collected in October as fresh green leaf, in December as autumnal leaf, and in April as dead leaf, respectively. Figure 1(d) shows the transient changes of the small parts of the fresh green leaf during heat treatment in phosphoric acid from 25 to 175 °C. The leaf tissue remained unchanged at 25 °C, started to melt or swell from the surface, and then was ruptured by swelling at 100 °C. A heating temperature of 150 °C is sufficient to obtain *M. sinensis* dissolved in phosphoric acid as a biohydrogen source for electrolysis. The fractions of cellulose and hemicellulose obtained from flax shives in phosphoric acid have been discussed in detail with respect to the acid concentration and treatment time^22^. The dissolution of cellulose and hemicellulose together with a gradual decrement of flax shives residue was observed in concentrated acid. Therefore, the dissolution of lignocellulosic materials was accelerated with an increase in the temperatures in this work.

### Continuous Electrolysis of *M. sinensis*

**Figure 2:**
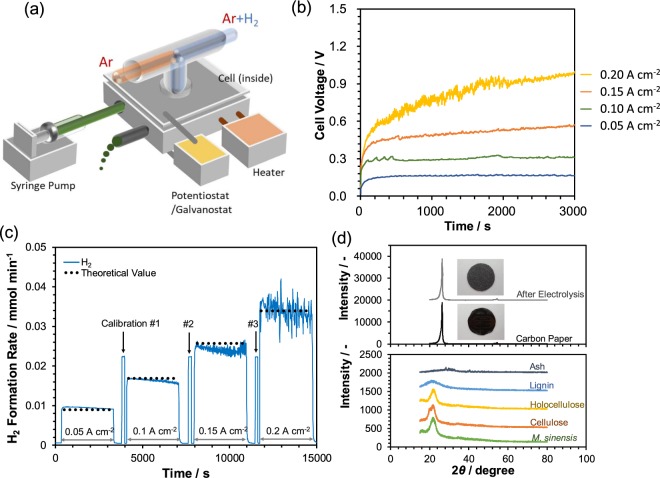
Electrolysis characteristics of the flow cell. (**a**) Illustration of the flow cell using *M. sinensis* as a biohydrogen resource. (**b**) Voltage-time curves and (**c**) rate of hydrogen formation during constant current operation at 150 °C. (**d**) XRD pattern of residues on the anode after continuous electrolysis compared with those of *M. sinensis* and its components.

Electrolysis using *M. sinensis* as a typical biohydrogen source was evaluated at 150 °C. To realize the continuous electrolysis of *M. sinensis*, the electrolysis cell shown in Fig. 2(a) was constructed. *M. sinensis* dissolved in phosphoric acid was introduced using a syringe pump at a flow rate of 0.44 mL min^−1^. The operating temperature of the cell was controlled such that the temperature of the anode side was kept at 150 °C using a cartridge heater. Figure 2(b) shows the transient change of the cell voltage for various constant current operations. Cell voltages at 0.05 and 0.1 A cm^−2^ were stable during constant current operation; however, the cell voltage did not become stable even after 3,000 seconds at 0.15 A cm^−2^. Furthermore, the cell voltage fluctuated up and down when operated at 0.2 A cm^−2^. The instability of the cell voltage, especially at high current densities, is considered to be due to the limitation on the migration of the *M. sinensis* solution to a reaction surface at the triple-phase boundary. At higher current density, higher diffusion rates of reactants and products are required for a proportionate electrochemical reaction to achieve a steady cell voltage. It should be emphasized that the observed cell voltage of 0.57 V for the electrolysis of *M. sinensis* at 0.15 A cm^−2^ was lower than those reported for methanol^23^ and ethanol electrolysis^24^. Another important aspect of the electrolysis of *M. sinensis* is the hydrogen production properties during long constant current operation. Figure 2(c) shows the experimental and theoretical hydrogen formation rates at current densities from 0.05 A cm^−2^ to 0.2 A cm^−2^ with intervals of 0.05 A cm^−2^ at 150 °C. Three calibration peaks for 1% hydrogen are also shown in Fig. 2(c). The hydrogen concentration in the outlet from cathode side increased when a constant current of 0.05 A cm^−2^ was applied to the electrolysis cell and reached the theoretical value calculated from following reactions for cellulose:

1 $${{\rm{C}}}_{6{\rm{n}}}{{\rm{H}}}_{10{\rm{n}}+2}{{\rm{O}}}_{5{\rm{n}}+1}+(7{\rm{n}}-1){{\rm{H}}}_{{\rm{2}}}{\rm{O}}\to 6{{\rm{nCO}}}_{{\rm{2}}}+24{{\rm{nH}}}^{+}+24{{\rm{ne}}}^{-}$$

2 $${{\rm{24nH}}}^{+}+{{\rm{24ne}}}^{-}\to {{\rm{12nH}}}_{2}$$

Therefore, the Faraday efficiency, which was estimated as the ratio of the actual hydrogen concentration to the theoretical value, was 1.01. The evolved hydrogen increased with the current density; however, it began to fluctuate when the current density exceeded 0.15 A cm^−2^, as shown in Fig. 2(b). This fluctuating behavior implies an intermittent release of hydrogen from the cathode, where protons receive electrons to form hydrogen molecules that then diffuse from the surface of the cathode catalyst to the argon carrier gas. The same phenomenon is reported for both water^25–27^ and water-methanol electrolysis^28^. Hydrogen bubbles generated on the surface of the cathode increase the reaction resistance by reducing the contact between the reactant and electrocatalyst, which inhibits electron transfer and results in an increase of ohmic resistance. The introduction of a “breather” for the release of generated gas to the outside has reduced the influence of the bubble effect in direct methanol fuel cells^29^. X-ray diffraction (XRD) measurements were performed to identify the residues on the anode surface after a long electrolysis experiment. The surface of the carbon electrode was partially covered with dark brown solids, as shown in the inset of Fig. 2(d). The XRD pattern for *M. sinensis* was equivalent to that previously reported^30^. No distinct peaks were observed for the residues on the carbon electrode except for those from the carbon fiber electrode made of graphite. Note that the absence of peaks due to *M. sinensis* and its derivatives implies not only the dehydration or degradation of *M. sinensis* but also the possibility of the occurrence of reaction (1) as the anode reaction.

The results indicate that *M. sinensis* has potential as a candidate biohydrogen source. Therefore, a batch type cell, where a sufficient amount of biomass material was supplied in advance to the anode side, was applied and analyzed to discuss the electrochemical reaction in the *M. sinensis* electrolysis cell in detail.

### Electrochemical properties of the cell using *M. sinensis*

**Figure 3:**
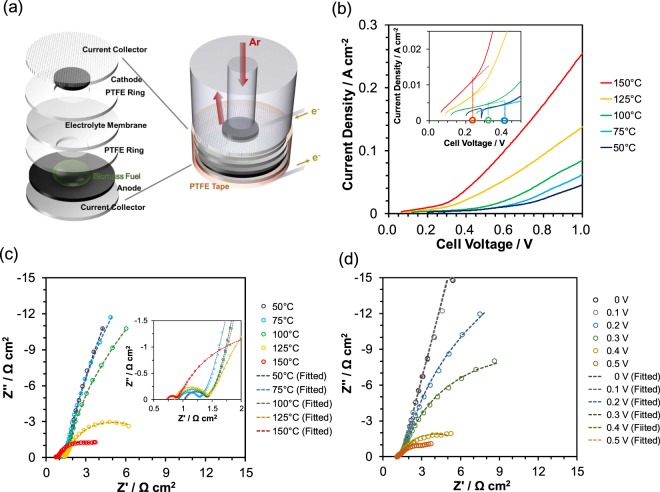
Electrolysis characteristics of the batch cell. (**a**) Illustration of the batch cell. (**b**) *I-V* curves and impedance spectra measured between 50 and 150 °C. (**d**) Impedance spectra measured at different applied voltages from 0 to 0.5 V at 150 °C.

Electrolysis using *M. sinensis* (and *P. lobata* and *S. altissima*) as a biohydrogen source was evaluated in the temperature range from 50 °C to 150 °C at 25 °C intervals. Figure 3(a) shows the components and structure of a batch type cell. Figure 3(b) shows the *I-V* characteristics of the electrolysis cell with *M. sinensis* at elevated temperatures. The open-circuit voltages in this temperature range were 0.06–0.25 V, which indicates that *M. sinensis* in phosphoric acid does not show significantly negative potentials versus Pt/C electrode in argon. The onset voltages for the electrolysis of *M. sinensis* decreased with an increase in temperature and reached a minimum of 0.24 V at 150 °C in the tested temperature range. The observed onset voltages are lower than those known for water electrolysis (ca. 1.5 V)^31,32^, equivalent to those for methanol electrolysis^33,34^, and similar to those reported for cellulose^14^, newspaper^15^, woody biomass^14^, and waste biomass^16^. The maximum current density at a cell voltage of 1.0 V increased with the temperature and reached a maximum of 0.254 A cm^−2^ at 250 °C, which is slightly lower than that for a cell with cellulose and equivalent to that with woody biomass^14^. The comparable onset voltage and *I-V* performance to other biomass materials reflect the main components of *M. sinensis* is holocellulose (58%), as shown in Fig. 1(b). Figure 3(c) shows impedance spectra for the cell under the same conditions as that for Fig. 3(b). The ohmic resistance decreased from 0.89 Ω cm^−2^ at 100 °C to 0.69 Ω cm^−2^ at 150 °C. This temperature dependence agrees well with the temperature dependence of the conductivity for a Sn_0.9_In_0.1_P_2_O_7_ electrolyte as previously reported^35^. The polarization resistance, which is composed of charge transfer resistance and mass transfer resistance, also decreased from 19.4 Ω cm^−2^ at 100 °C to 2.1 Ω cm^−2^ at 150 °C, which suggests that the reduction of polarization resistance accounts for the improvement of the *I-V* performance and resistivity. Below 100 °C, the operating temperature is not sufficiently high to directly electrolyze cellulose or related components; therefore, the direct oxidation of cellulose does not occur readily, but indirect oxidation reaction or other oxidation reactions may occur. Figure 3(d) shows impedance spectra for the cell at various applied cell voltages and 150 °C. The ohmic resistance was not dependent on the applied voltage; however, the polarization resistance was heavily dependent on the applied voltage, especially over 0.4 V. This tendency corresponds to the *I-V* profiles shown in Fig. 3(b). The decrease in polarization resistance over 0.4 V supports that *M. sinensis* was electrolyzed to hydrogen at less than 0.4 V. Similar dependence of impedance spectra on the applied voltage has been reported for methanol and water electrolysis^36^, and ethanol-water electrolysis^37^. Electrochemical reactions were accelerated with an increase in the applied voltage and with the same ohmic contributions. Here, the onset voltage of *M. sinensis* electrolysis at 150 °C was calculated to be 0.24 V. The electrolysis performance of the cells using *M. sinensis* collected during different seasons and from different places is shown in Fig. S2. There was no distinct difference in the *I-V* curves, which suggests there is little difference among the tested *M. sinensis* as biomass resources. To evaluate the applicability of other plant species, *P. lobata* and *S. altissima* were also examined using the same procedure as that for *M. sinensis*. The electrolysis performance of these plant species are summarized in Fig. S3. No major difference was observed between *P. lobata* and *S. altissima*, which indicates that they can both be used as weedy biomass resources for hydrogen production. Thus, *P. lobata* could be selected if climbing plants are suitable for a cultivated land^38^, and *M. sinensis* if the environment is temperate throughout the year.

### Hydrogen production from *M. sinensis*

**Figure 4:**
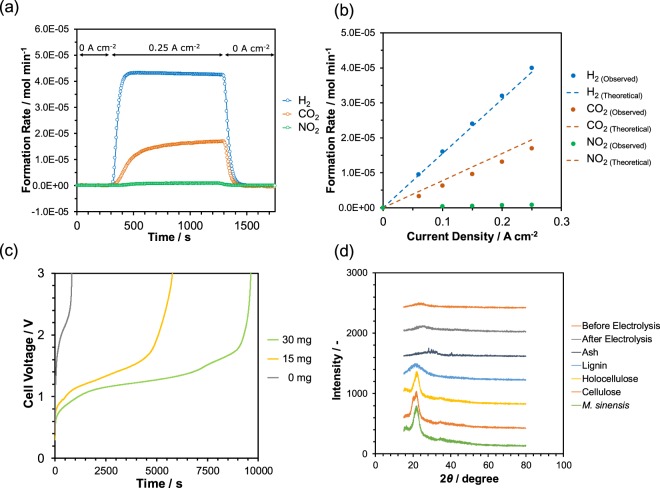
Hydrogen production. Evolution and formation rates of (**a**) hydrogen, carbon dioxide, and nitrogen dioxide during current operation, and (**b**) dependence of formation rates on current density at 150 °C. Theoretical values are included for comparison. (**c**) Cell voltage during constant current operation for various amounts of *M. sinensis* at 150 °C. (**d**) XRD pattern of residue on the anode after constant current operation and XRD patterns for *M. sinensis* and its components.

Hydrogen production properties were evaluated for the electrolysis of *M. sinensis* at 150 °C. Figure 4(a) shows the formation rates of hydrogen, carbon dioxide, and nitrogen dioxide during constant current operation (0.25 A cm^−2^). Stable hydrogen formation was achieved for 1,000 seconds of constant current operation. Figure 4(b) shows the observed and theoretical (according to Eqs (1–3)) formation rates of hydrogen, carbon dioxide, and nitrogen dioxide with respect to a current density.

Reaction of protein:

3 $${\rm{C}}\,{\rm{or}}\,{\rm{N}}\,{\rm{in}}\,{\rm{fuel}}+{\rm{2O}}\,{\rm{in}}\,{\rm{fuel}}\,{\rm{and}}\,{{\rm{H}}}_{{\rm{2}}}{\rm{O}}\to {{\rm{CO}}}_{{\rm{2}}}\,{\rm{or}}\,{{\rm{NO}}}_{{\rm{2}}}+{{\rm{4H}}}^{+}+{{\rm{4e}}}^{-}$$

*M. sinensis* contains 5% of protein as crude protein; therefore, nitrogen dioxide is possibly produced through electrolysis at the anode^39^. The rate of hydrogen formation increased with the current density and was equivalent to the theoretical values, which implies that reaction (1) occurred during the electrolysis of *M. sinensis*, with high Faraday efficiency of approximately 1. On the other hand, the formation rate of carbon dioxide was slightly lower than theoretical (Faraday efficiency: 0.87 at 0.25 A cm^−2^) and the formation of nitrogen dioxide was confirmed instead (Faraday efficiency: 0.04 at 0.25 A cm^−2^). Not all the active oxygen generated through electrolysis was used to oxidize carbon in the cellulosic component, but was also used to oxidize nitrogen in the protein component according to reaction (3). Figure 4(c) shows the time dependence of the cell voltage for the cells containing different amounts of *M. sinensis* at the anode side during constant current operation at 0.1 A cm^−2^. For each electrolysis cell, plateau voltages continued below 1.4 V and stable cell voltages were observed; however, after reaching 1.4 V or higher, the cell voltage began to rapidly increase until over the cutoff voltage of 3.0 V. The lengths of the plateau times were proportional to the weight of *M. sinensis*, which indicates that *M. sinensis* was consumed during constant current operation, and electrolysis would continue if *M. sinensis* remained at the anode side. Thus, the residues at the anode side after long constant current operation are by-products that were non-electrolyzable solids. Figure 4(d) shows XRD patterns of the anode surfaces before/after electrolysis at a constant current of 0.1 A cm^−2^ until the cell voltage exceeded the cutoff voltage of 3.0 V, in addition to those of *M. sinensis* components, such as holocellulose, cellulose, lignin, extractives, and ash. After long time electrolysis, distinctive peaks due to *M. sinensis* and other components were not observed, which indirectly shows the dissolved *M. sinensis* in phosphoric acid was consumed to form hydrogen and carbon dioxide with residues in untraceable forms such as in solution or as amorphous phases.

### Discussion on the electrolysis of *M. sinensis*

**Figure 5:**
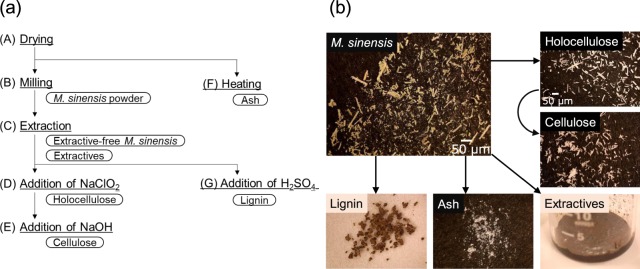
Separation of *M. sinensis* components. (**a**) Preparation procedure and (**b**) photographs of the *M. sinensis* components.

**Figure 6:**
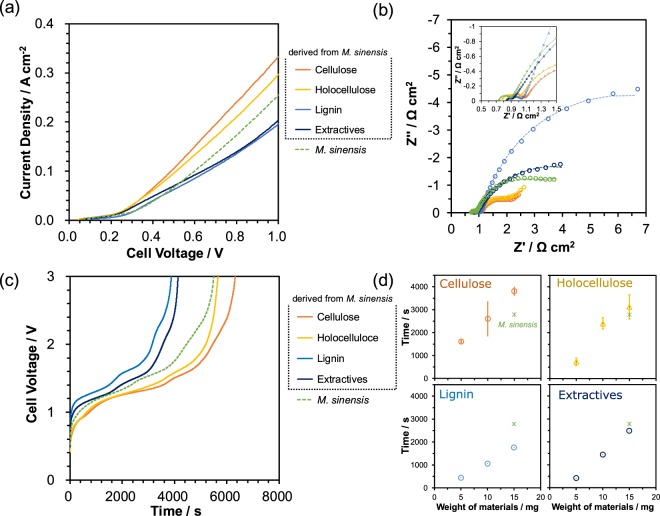
Electrolysis characteristics for the batch cell using *M. sinensis* components as biohydrogen resources. (**a**) *I-V* curves and (**b**) impedance spectra for the batch cells using *M. sinensis* components at 150 °C. (**c**) Voltage-time curves for the batch cells at a constant current density of 0.1 A cm^−2^ and 150 °C. (**d**) Dependence of the electrolysis time (<1.5 V) on the amount of biomass from 5 to 15 mg.

The results reveal that *M. sinensis* swelled and was dissolved in phosphoric acid, and can be electrolyzed from a low onset voltage of 0.24 V with sufficient current density to form hydrogen at a rate that follows the complete electrochemical oxidation of cellulose. Therefore, *M. sinensis* has potential as a candidate biohydrogen resource. Details of *M. sinensis* electrolysis has not become clear, therefore, electrochemical properties of several *M. sinensis* components such as holocellulose, cellulose, lignin, extractives, and ash were evaluated. The procedure to separate each component from *M. sinensis* is summarized in Fig. 5(a) and each component is shown in Fig. 5(b). The *M. sinensis* components obtained were powdery or polycrystalline, except for the extractives, which were a thick dark-brown paste. The *I-V* characteristics of the electrolysis cells using each of the *M. sinensis* components are shown in Fig. 6(a) together with that for *M. sinensis*. The *I-V* curves for holocellulose and cellulose had the same onset voltage of 0.22 V and were improved with respect to that of *M. sinensis*. In contrast, the *I-V* curves for lignin and the extractives showed lower current densities than those of holocellulose and cellulose at 1.0 V. For lignin, the onset voltage was shifted to 0.25 V, which implied the relative inactivity of lignin. The same tendency was observed in the impedance measurements shown in Fig. 6(b). The impedance spectra suggest that the *I-V* characteristics reflect the order of polarization resistance during electrolysis. The relatively large polarization resistance of lignin for electrolysis has been reported previously^40^. Note that lignin in this experiment is referred to as a Klason lignin, which is obtained as a residue from the hydrolysis of lignocellulosic materials and is partially altered using concentrated sulfuric acid at elevated temperature and high pressure^41^. Lignin is relatively stable in acid at high temperatures because of this acid treatment; therefore, it may exhibit a large polarization resistance. Figure 6(c) shows the transient changes of the cell voltages during constant current operation at 0.1 A cm^−2^. The amount of biomass source was fixed at 15 mg. Lignin showed the shortest duration time among the tested samples, which also accounts for the highest polarization resistance observed for lignin cause by the relatively high cell voltage (Fig. 6(c)). The order of the duration times was cellulose > holocellulose > lignin = extractives, which indicates that the components with high *I-V* performance charcteristics (cellulose and holocellulose) are easily electrolyzed to hydrogen and oxidized to carbon dioxide at low cell voltages. This tendency continued with proportionality to the biomass weight when the content of each component in the cells was changed from 5 to 15 mg, as shown in Fig. 6(d). The difference in the *I-V* and impedance characteristics, and the duration times of cellulose and holocellulose is summarized as follows. Hemicellulose, which is one of the components of holocellulose, is generally composed of polysaccharides other than glucose (see Supplementary Table 1 and previous works^19,42^). The materials from *M. sinensis* dissolved in phosphoric acid at 150 °C were analyzed using liquid chromatography-mass spectrometry (LC-MS). The chemical compositions of the detected molecules were mainly C_5_H_7_O_3_ and C_6_H_5_O_6_, which indicates that hemicellulose can be hydrolyzed to derivatives of constituent sugars at the operation temperature of 150 °C. In contrast, no glucose or other related hydrolysis products were detected, which indicates that lignocellulose was just swelled or dissolved in phosphoric acid. Therefore, the loss of the hemicellulose caused a deterioration in the electrolysis performance at 150 °C. Holocellulose remained as ordered fibers, as observed in the original structure; however, cellulose was partially degraded in the structure after the separation treatment (see Fig. S4). Both lignin and the remaining hemicellulose help to construct a fibrous structure, which results in comparative stability in phosphoric acid. The results and observations described here suggest the observed electrolysis performance of *M. sinensis* is reasonable because the *I-V*, impedance, and constant current performance are between those for cellulose and lignin.

## Conclusions

As a biomass material that is not typically utilized, *M. sinensis* was tested as a biohydrogen source for the direct electrolysis of lignocellulose at an intermediate temperature of 150 °C. The electrolysis of *M. sinensis* is characterized as follows: onset voltage and maximum current density (at 1.0 V) for electrolysis were 0.24 V and 0.254 A cm^−2^, respectively. The current efficiency for hydrogen production was approximately 1.0 at several constant current densities. For the typical components of *M. sinensis*, the order of electrolysis effectiveness was cellulose > holocellulose > extractives = lignin. Lignin and the ordered and well-structured fibrous tissue of delignified *M. sinensis* is responsible for the relative stability in the acid solvent, which results in lowering of the effectiveness of electrolysis. The inclusion of undesirable components at this stage is unavoidable for the direct electrolysis of weeds; however, the applicability of weeds as a biohydrogen source will lighten the dependence on oil and accelerate the development of biomass technology.

## Methods

### Materials

An electrolyte membrane that provides proton conduction and separation between electrodes was prepared by mixing Sn_0.9_In_0.1_P_2_O_7_ powder (1.0 g) with polytetrafluoroethylene (PTFE) powder (0.04 g), followed by cold-rolling the mixture to form a thin film (200 μm thick). Commercially available Ketjenblack (KB; EC-600JDK) was modified as follows. KB (1.0 g) was added in 24% HNO_3_ (50 mL) and stirred at room temperature for 3 days. After filtering and washing with deionized water until the filtrate indicate neutral, the carbon was dried under reduced pressure at 120 °C for 6 h, followed by the heat treatment at 600 °C for 5 h in a flow of argon. The obtained carbon (0.1 g) was added to 85% H_3_PO_4_ (0.9 g) and mixed in a mixer (Thinky AR-100) for 5 min. The obtained mixture was spread on the carbon paper, where the amount of carbon was adjusted to 10 mg cm^−2^. A commercially available Pt/C electrode (Pt loading: 2 mg cm^−2^, Electrochem) was used as a cathode. *M. sinensis* was gathered in October, December 2017, and March 2018 at Nagoya in Japan, and in December 2017 at Okinawa in Japan. Leaves of *M. sinensis* were cut into small pieces (4–6 mm) for air-drying overnight. The cut and dried leaves were milled to a powder with a grinder (Wonder Blender, WB-1) and stored in a desiccator. Other weeds such as *Pueraria lobata* (*P. lobata*) and *Solidago altissima* (*S. altissima*), which represent climbing plants and perennial plants, respectively, were examined to compare the difference between species. Both *P. lobata* and *S. altissima* were prepared by applying the same procedure as that used for *M. sinensis*.

### Characterization of *M. sinensis*

The chemical composition of *M. sinensis* was determined using commonly used procedures. Briefly, air-dried and milled *M. sinensis* was incinerated at 600 °C to calculate ash content. Components soluble in alcohol and benzene were extracted using a Soxhlet extractor. Holocellulose was separated by alkaline treatment using sodium chlorite. Holocellulose was further purified to obtain cellulose by alkaline treatment using sodium hydroxide. The amount of hemicellulose was calculated from the difference in the amount between holocellulose and cellulose. Acid-insoluble lignin was separated from extracted powder using concentrated sulfuric acid, followed by autoclave treatment at 120 °C. The amount of acid-soluble lignin was calculated from the UV absorbance (210 nm) of the filtrate. Starch was determined by the enzyme method. Crude protein was determined by multiplying the nitrogen content measured using a CHN analyzer by 6.25. The chemical composition of the saccharides and derivatives in the filtrate from *M. sinensis* immersed in with 85% phosphoric acid at 150 °C was determined using high-performance liquid chromatography with tandem mass spectrometry (LC-MS; Shimadzu LC-30AD). The amounts of hydrogen and carbon dioxide produced from the electrodes were monitored with a mass spectrometer (MS; Pfeiffer Vacuum ThermoStar). The morphology of *M. sinensis* and its derivatives were observed using an optical camera and scanning electron microscopy (SEM; Keyence VE-8800). X-ray diffraction (XRD) patterns of *M. sinensis* and its derivatives were recorded on an X-ray diffractometer (Rigaku, MiniFlex II).

### Electrochemical measurements

Two types of electrolysis cells were fabricated for electrochemical measurements^14,15^. One was a flow type cell and the other was a batch type cell. The same anode (12 mm diameter), cathode (8 mm diameter), and electrolyte membrane (16 mm diameter) were applied for both cells. The flow cell mainly consisted of stainless-steel plates with flow channel, a separator, and cartridge heaters. A mixture of *M. sinensis* and 85% phosphoric acid (*M. sinensis* concentration: 0.35 wt%) was continuously injected to the anode side at a flow rate of 0.44 mL min^−1^ using a syringe feeder. The cathode was supplied with argon and the outlet was connected to a mass spectrometer to monitor the generated gases. For the batch cell, *M. sinensis* powder (15 mg) was impregnated with 85% phosphoric acid (ca. 34.4 μL) and the mixture (thickness: < 1 mm) was spread on the anode surface, and attached on to a current collector, followed by the sealing with PTFE tape. Argon (100 mL min^−1^) was supplied to the cathode. Electrochemical data such as *I-V* curves and impedance spectra were collected using an electrochemical interface (Solartron 1287) and frequency response analyzer (Solartron 1260). Potentiodynamic measurements were performed at a scan rate of 20 mV s^−1^ in the voltage range from open-circuit to 1.0 V. Galvanostatic measurements were performed in the current density range of 0.05–0.25 A cm^−2^. Impedance spectra were recorded at a bias voltage of 0.0–0.4 V in the frequency range of 0.1–10^6^ Hz.

## Electronic supplementary material

SUPPLEMENTARY INFO
