# Supplementary material for: Intermediate-temperature electrolysis of energy grass Miscanthus sinensis for sustainable hydrogen production
Source: Sci Rep. 2018 Nov 1;8:16186. doi: 10.1038/s41598-018-34544-y (PMC6212540; doi:10.1038/s41598-018-34544-y)
Supplement: Supplementary file 1 — SUPPLEMENTARY INFO [file 41598_2018_34544_MOESM1_ESM.docx]

Supplementary Information

**Intermediate-temperature electrolysis of energy grass *Miscanthus sinensis* for sustainable hydrogen production**

Masaya Ito ^a^, Tetsuya Hori ^b^, Shinya Teranishi ^b^, Masahiro Nagao ^a*^, Takashi Hibino ^a^

^a^ Graduate School of Environmental Studies, Nagoya University, Nagoya 464-8601, Japan

^b^ SOKEN Inc., Nisshin, Aichi 470-0111, Japan

^*^ E-mail: nagao@urban.env.nagoya-u.ac.jp

Table of Contents

Figs. S1–4

Table S1

Holocellulose, cellulose, lignin, ash, and extractives prepared from *M. sinensis*

**Fig. S1.** Photographs of (a) *Pueraria lobata* and (b) *Solidago altissima*.

1. (b)

**Fig. S2.** *I-V* curves for electrolysis cells using *M. sinensis* gathered from (a) Nagoya and Okinawa, and (b) gathered in October, December 2017 and April 2018 from Nagoya.

**Fig. S3.** (a) *I-V* curves and (b) impedance spectra for electrolysis cells using *P. lobata*, *S. altissima*, and *M. sinensis* at 150°C.

**Fig. S4.** Micrographs of (a) holocellulose after delignification, and (b) cellulose after delignification and purification of *M. sinensis*.

**Table S1.** Constituent sugars of *M. sinensis* gathered from Nagoya in October 2017.

| Monosaccharide | Molecular Formula | %, dry weight basis |
| --- | --- | --- |
| Glucose | C_6_H_12_O_6_ | 32.2 |
| Xylose | C_5_H_10_O_5_ | 14.7 |
| Arabinose | C_5_H_10_O_5_ | 3.2 |
| Fructose | C_6_H_12_O_6_ | 2.6 |
| Galactose | C_6_H_12_O_6_ | 1.2 |
| Rhamnose | C_6_H_12_O_5_ | 0.7 |
| Mannose | C_6_H_12_O_6_ | 0.2 |

**Holocellulose, cellulose, lignin, ash, and extractives prepared from *M. sinensis***

Leaves of *M. sinensis* were cut into small pieces for air-drying overnight. The cut and dried leaves were milled to a powder with a grinder (Wonder Blender, WB-1 Osaka Chemical Co., Ltd) and stored in a desiccator. The leaf water content was determined by weighing before and after drying in a drying machine at 105°C for 2 h. The leaf ash content was calculated from the weight of residue after ignition at 600°C for 2 h. The ethanol and benzene soluble fractions were collected by Soxhlet extraction. A milled leaf powder (10 g) was extracted continuously for 6 h with ethanol-benzene (1:2 by volume ratio) and 4 h with ethanol in a Soxhlet extractor. The extractive-containing solution was dried at 60°C in a vacuum drying machine. Holocellulose was separated from extractive-free leaf by the delignification method with sodium chlorite/acetic acid (Wise et al.). Briefly, extractive-free leaf powder (2.5 g) was dispersed in deionized water (150 mL) followed by the addition of sodium chlorite (1.0 g) and acetic acid (0.2 mL). The mixture was placed on a heater at 80°C for 1 h in a 300 mL beaker. The addition of sodium chlorite and acetic acid, and the heating process were repeated twice. After a total of 3 h delignification, the mixture was filtered with a glass filter and washed with deionized water (2 L) until the filtrate became neutral. The residue was then washed with acetone (50 mL) and placed in a drying machine at 105°C until the weight became constant. The holocellulose was further purified to obtain cellulose. Holocellulose was dispersed in 17.5% aqueous sodium hydroxide (25 mL) at 20°C for 30 min followed by the addition of deionized water and stirring for 1 min. After leaving at 20°C for 5 min, the residue was filtered with a glass filter and washed with deionized water (2 L) and 10% acetic acid (40 mL) followed by washing with boiled water (1 L) to complete the purification. As in the case with holocellulose, the purified cellulose was also placed in a drying machine at 105°C until the weight became constant. Starting from the extractive-free leaf powder, lignin (Klason lignin) was also derived through the acid treatment. 72% sulfuric acid (3 mL) was added to extractive-free leaf powder (300 mg) and left at 30°C for 1 h. Deionized water was then added to the mixture and autoclaved at 120°C for 1 h. The obtained mixture was filtered with a glass filter while it was hot for analysis of the acid-soluble lignin in the filtrate. The residue on the glass filter was gently washed with deionized water and dried at 105°C until the weight became constant. The concentration of the acid-soluble lignin in the filtrate was determined by measuring the UV absorbance at 210 nm.
